# Royal Jelly-Loaded LDH Nanostructures: A Novel Topical Therapy for Atopic Dermatitis via Inflammasome Suppression in Mice

**DOI:** 10.34172/apb.46710

**Published:** 2026-06-27

**Authors:** Fardin Khajepour, Mehdi Ranjbar, Merat Mahmoodi, Nasrin Bazargan, Fatemeh Mohammadi Henjeroei, Muhammad Hossein Ashoub, Reza Nosratabadi

**Affiliations:** ^1^Student Research Committee, Afzalipour Faculty of Medicine, Kerman University of Medical Sciences, Kerman, Iran; ^2^Pharmaceutics Research Center, Institute of Neuropharmacology, Kerman University of Medical Sciences, Kerman, Iran; ^3^Department of Medical Immunology, Afzalipour Faculty of Medicine, Kerman University of Medical Sciences, Kerman, Iran; ^4^Department of Pediatrics, Afzalipour Medical Center, Afzalipour Faculty of Medicine, Kerman University of Medical Sciences, Kerman, Iran; ^5^Department of Hematology and Medical Laboratory Sciences, Faculty of Allied Medicine, Kerman University of Medical Sciences, Kerman, Iran; ^6^Gastroenterology and Hepatology Research Center, Kerman University of Medical Sciences, Kerman, Iran

**Keywords:** Atopic dermatitis, Royal jelly, Layered double hydroxide nanostructures, Anti-inflammatory, Topical delivery

## Abstract

**Purpose:**

Atopic dermatitis (AD) is a chronic autoimmune disease of the skin, resulting from immune dysregulation and inflammasome activation. This study aimed to develop and evaluate a topical formulation of layered double hydroxide (LDH) nanostructures loaded with royal jelly (RJ) for its therapeutic effects on AD in a mouse model.

**Methods:**

LDH nanostructures were synthesized, and characterized by nanoparticle specific tests. The RJ-loaded nanostructures were incorporated into a carbomer-based gel, and sustained release of RJ was measured using a Franz diffusion cell. AD was induced in female BALB/c mice using 2, 4-dinitrochlorobenzene (DNCB)-induced AD. The mice were then divided into six groups: control, Free-RJ (100 mg/kg), Free-LDH, and RJ-loaded LDH at different doses (50, 100, and 200 mg/kg).

**Results:**

Treatment with RJ-loaded LDH nanostructures significantly diminished the expression of inflammasome-related genes (NLRP3, Caspase-1, IL-1β, and IL-18) in spleen cells in a dose-dependent manner. Serum levels of IL-1β, IL-18, and IgE were also significantly decreased, and histopathological analysis revealed reduced epidermal thickening and inflammation in the treated groups.

**Conclusion:**

These findings suggest that RJ-loaded LDH nanostructures enhance RJ’s anti-inflammatory and immunomodulatory effects, offering a promising topical therapeutic strategy for AD management.

## Introduction

 Atopic dermatitis (AD) is a chronic, relapsing inflammatory skin disorder, affecting approximately 20% of children and 3–5% of adults worldwide, imposing a significant burden on quality of life and healthcare systems.^1,2^ The pathogenesis of AD is multifactorial, involving genetic predispositions, environmental triggers, and immune dysregulation.^3^ A hallmark of AD is the activation of the NLRP3 inflammasome, a multiprotein complex that orchestrates the innate immunity by activating Caspase-1, leading to the maturation and release of pro-inflammatory cytokines such as IL-1β and IL-18.^4-6^ These cytokines perpetuate a cycle of inflammation, exacerbating skin barrier dysfunction, and pruritus, as well as immune cell infiltration in AD lesions.^7,8^

 Royal jelly (RJ), produced by worker honeybees, has emerged as a natural therapeutic agent due to its anti-inflammatory, antioxidant, and immunomodulatory properties.^9,10^ RJ contains major bioactive compounds, including 10-hydroxy-2-decenoic acid (10-HDA), proteins, and peptides that have shown potential in alleviating inflammatory conditions, including dermatitis.^11,12^ However, the clinical application of RJ is hindered by its poor stability, low bioavailability, long-term pharmacotherapy, and rapid degradation under physiological conditions, necessitating advanced delivery systems to enhance its therapeutic efficacy.^13,14^

 Nanotechnology offers a promising solution to these challenges by improving the stability, bioavailability, high loading efficiency, and targeted delivery of bioactive compounds.^15^ LDH nanostructures, often referred to as anionic clays, are biocompatible materials with a layered structure that can intercalate and release bioactive molecules in a controlled manner.^16,17^ Their high surface area, tunable properties, and ability to protect encapsulated compounds make them ideal carriers for topical drug delivery in dermatological disorders.^18,19^ Previous studies have also documented the efficacy of LDH-based systems in delivering anti-inflammatory agents, such as curcumin and dexamethasone, for the treatment of skin disorders.^20-23^

 In the current study, we aimed to develop a topical formulation of RJ-loaded LDH nanostructures and evaluate its effects on inflammasome activation and downstream inflammatory pathways in a DNCB-induced AD mouse model to provide a comprehensive evaluation of the nanoformulation’s potential as a novel therapeutic strategy for AD.

## Materials and Methods

###  Materials

 Aluminum silicate, magnesium, sodium hydroxide (NaOH), carbomer, and DNCB were purchased from Sigma-Aldrich (St. Louis, MO, USA). Royal jelly was obtained from a local apiary (Kerman, Iran) and stored at -20°C until use. RPMI medium, RNA Extraction Kit was sourced from Ratin Gene, Iran and SYBR Green Master Mix was purchased from Parstous, Iran. ELISA kits for IL-1β, IL-18, and IgE were obtained from Karmania Pars Gene Company, Kerman, Iran. All other chemicals were of analytical grade.

###  Synthesis of LDH Nanostructures

 LDH nanostructures were synthesized using a co-precipitation method. Briefly, 0.05 g of aluminum silicate and 0.05 g of magnesium were weighed and mixed in a 100 mL round-bottom flask, followed by adding 10 mL of distilled water. The mixture was stirred at 50°C for 30 minutes at 400 rpm on a magnetic stirrer. Subsequently, 3–4 drops of 1M NaOH were added to adjust the pH, and the flask was connected to a reflux system. Then the mixture was stirred for 30 minutes at 50°C and 400 rpm. After cooling, the solution was subjected to ultrasonication at 60 W for 10 minutes using an ultrasonic bath, followed by microwave treatment at 180 W for 10 minutes in a microwave oven, with intermittent pauses every 30 seconds to prevent boiling. A portion of the synthesized nanoparticles was separated for quality control tests, and the remainder was stored at 4°C.^24^

###  Loading of Royal Jelly into LDH Nanostructures

 Royal jelly was loaded into the LDH nanostructures using the co-precipitation method. The nanoparticle suspension was shaked by a magnetic stirrer at 50°C and 400 rpm, connected to a reflux system to facilitate nucleation. Royal jelly was added to achieve final concentrations of 50, 100, and 200 mg/kg, calculated based on an average mouse weight of 20 g and a 100 µL administration volume. The mixture was stirred for 30 minutes to allow RJ to intercalate between the nanoparticle layers during the nucleation process.

 To determine how much RJ was loaded in LDH nanostructures, the encapsulation efficiency value calculated using UV–Vis spectrophotometer at 260 nm in ABS. 0.9 in the presence of ethanol 96% and buffer phosphate 7.2. The encapsulation efficiency was calculated about 99.88%. The drug entrapment efficiency Royal Jelly in nanostructures were evaluated according to below equation.

 Entrapment efficiency (EE) % = (Wt/Wi) × 100%.

 Where Wt is the total amount of the incorporated RJ and Wi is the total quantity of incorporated RJ added initially during the preparation process. *In vitro* dapsone release from polyacrylamide/polylactic acid core/shell nanofibers was determined by UV-spectrometer S-3100 SCINCO.

###  Characterization of Nanostructures

 The size distribution and zeta potential of the nanoparticles were determined using Dynamic Light Scattering (DLS) with a Malvern Zetasizer Nano ZS (Malvern Instruments, UK) at a wavelength of 633.8 nm, 20°C, and a scattering angle of 173°. Each sample was analyzed in triplicate. The morphology of the nanoparticles was examined using Scanning Electron Microscopy (SEM) (Philips EM208S, Germany) at the Razi Metallurgical Research Center (Tehran, Iran). Nanostructures were prepared by diluting 5 mL of the nanoparticle suspension, drying on a carbon-coated grid, and imaging at an accelerating voltage of 20 kV. Fourier Transform Infrared Spectroscopy (FTIR) (Perkin-Elmer Spectrum 100, USA) was performed in the range of 400–4000 cm⁻¹ with a scan speed of 2 cm⁻¹ to identify functional groups and interactions between LDH and RJ. Nanostructures were centrifuged at 14,000 g for 30 minutes, washed three times with deionized water, dried at 60°C for 24 hours, and ground into a powder for analysis.^25^

###  Preparation of Gel Formulation

 The RJ-loaded nanoparticle suspension was converted into a gel using a carbomer as a gelling agent. The suspension was stirred at 50°C and 400 rpm on a magnetic stirrer. At the same time, carbomer powder was gradually added at a concentration of 0.001 g/ml to increase gel stability and adjust to a concentration of 8 g/ml to reach saturation. This is due to the high stability in the manufacture of the final product and the creation of a stable composition in the final product, which is defined as a non-flowing gel when applied to a gloved hand. The gel was stored at 4°C until use.

###  In Vitro Release Study

 The release of RJ from the gel formulation was evaluated using a Franz diffusion cell (PermeGear, USA) with a cellulose acetate membrane (MW cutoff = 12,000 Da, Visking Dialysis Tube). The receptor compartment contained 15 mL of normal saline maintained at 37 ± 1°C using a circulating water bath. This cell had two parts, an acceptor and a donor, between which a suitable membrane made of cellulose acetate. One milliliter of the gel formulation was placed in the donor compartment, and the membrane was pre-soaked in saline for 24 hours to stabilize its thickness.^26^

 Samples (1 mL) were withdrawn from the receptor compartment at 0, 15, 60, 90, 120, 150, 180, and 240 minutes, and the volume was replaced with fresh saline to maintain sink conditions. The RJ concentration was measured using a UV-Vis spectrophotometer (Shimadzu, Japan) at 240 nm.

###  Animal Model and Experimental Design

 Thirty-six BALB/c mice (female, 6–8 weeks-old, 18–22 g) were purchased from the animal facility of Kerman University of Medical Sciences (Kerman, Iran) and housed under standard conditions (22 ± 2°C, 12-hour light/dark cycle, ad libitum access to food and water). In this study hair removal was done with electrical clippers, and followed by using a chemical depilatory agent for further hair removal. Then, AD was induced by applying 200 µL of 4% DNCB (dissolved in acetone: olive oil, 4:1) to the dorsal skin (between the neck and scapulae) on days 1 and 4 for sensitization, followed by 200 µL of 0.4% DNCB on days 7 and 11 for challenge.

 The mice were randomly divided into six groups (n = 6 per group):

- **Group 1 (Control, Ctrl):** AD was induced, and received an acetone/olive oil mixture [4:1 (v/v)].
- **Group 2 (Free-RJ):** AD was induced, and treated with Free-RJ (100 mg/kg).
- **Group 3 (Free-NP):** AD was induced, and treated with unloaded LDH (100 mg/kg).
- **Group 4 (NP-RJ-50):** AD was induced, and treated with RJ-loaded LDH (50 mg/kg).
- **Group 5 (NP-RJ-100):** AD was induced, and treated with RJ-loaded LDH (100 mg/kg).
- **Group 6 (NP-RJ-200):** AD was induced, and treated with RJ-loaded LDH (200 mg/kg).

Treatment was administered topically (100 µL) once daily from day 12 to day 28. The research was approved by the Ethics Committee of Kerman University of Medical Sciences (The ethical approval code: IR.KMU.AH.REC.1399.175, approval number 99000771), and all procedures adhered to international guidelines for the ethical use of animals in research.

###  Sample Collection

 On day 28, mice were anesthetized and sacrificed as per animal ethics. Blood was collected from the heart using a syringe, and the sera were separated by centrifugation at 3000 rpm for 5 minutes at 4°C. Spleen cells were isolated under a laminar flow hood by injecting 5 mL of cold RPMI medium into the spleen, followed by centrifugation at 2500 rpm for 10 minutes. Red blood cells were lysed using RBC Lysis Buffer for 10 minutes at 4°C, and the remaining cells were washed with PBS, resuspended in 2 mL of RPMI, and counted using a Neubauer chamber after trypan blue staining. Skin samples from the dorsal region were fixed using 10% neutral buffered formalin for histopathological analysis.

###  Gene Expression Analysis

 Spleen cells (5 × 10⁶) were used for RNA extraction using a Total RNA Extraction Kit (Ratin Gene, Iran). 1µg of RNA was reverse-transcribed into cDNA using a cDNA synthesis kit (Ratin Gene, Iran). The expression level of NLRP3, Caspase-1, IL-1β, IL-18, and β-actin (housekeeping gene) genes was analyzed using Real-Time PCR with RealQ Plus 2x Master Mix Green (Parstous, Iran) on an Applied Biosystems StepOnePlus system (Thermo Fisher Scientific, USA). Primer sequences are listed in Table 1. The reaction conditions were as follows: initial denaturation; 95°C for 10 minutes, followed by 40 cycles of 95°C for 15 seconds and 60°C for 1 minute. Relative gene expression was calculated using the 2^-ΔΔCt^ method, normalized to β-actin.^27^

**Table 1:** Primer sequences for Real-Time PCR.

**Gene**	**Primer Sequence (5’→3')**	**Length (nt)**
IL-1β Forward	TTCAGGCAGGCAGTATCACTC	21
IL-1β Reverse	GAAGGTCCACGGGAAAGACAC	21
IL-18 Forward	CCCACGCTTTACTTTATACCTGA	23
IL-18 Reverse	CCCTTTTGTCAACGAAGAGAAC	22
NLRP3 Forward	ATTACCCGCCCGAGAAAGG	19
NLRP3 Reverse	TCGCAGCAAAGATCCACACAG	21
Caspase-1 Forward	ACAAGGCACGGGACCTATG	19
Caspase-1 Reverse	TCCCAGTCAGTCCTGGAAATG	21
β-actin Forward	CACTGTCGAGTCGCGTCC	18
β-actin Reverse	TCATCCATGGCGAACTGGTG	20

###  Cytokine and IgE Measurement

 Serum levels of IL-1β, IL-18, and IgE were measured using commercial ELISA kits (Karmania Pars Gene Company, Kerman, Iran) based on the sandwich ELISA method. Fifty microliters of serum were added to each well, and the assay was carried out according to the manufacturer’s protocol. Optical density was measured at 450 nm using a microplate reader (BioTek, USA).

###  Histopathological Analysis

 Skin samples were processed, then embedded in paraffin, sectioned at 5 µm, and stained with hematoxylin and eosin (H&E). Slides were examined using a light microscope (Olympus, Japan) at 20 × and 40 × magnifications to assess epidermal thickness, hyperkeratosis, and inflammatory cell infiltration.

###  Statistical Analysis

 Data were expressed as mean ± SEM. Differences between groups were analyzed using one-way ANOVA followed by Tukey’s post-hoc test in SPSS 19 (IBM, Armonk, NY, USA). Statistical significance was defined as *P < 0.05, **P < 0.01, and ***P < 0.001.

## Results

###  Characterization of LDH Nanostructures and Royal Jelly Loading

 The synthesis of LDH nanostructures was successfully achieved, as confirmed by various analytical techniques. Dynamic Light Scattering (DLS) analysis indicated that the average particle size of the synthesized free LDH (Free-NP) nanostructures was 61.8 nm (Figure 1A), with a relatively narrow size distribution. After loading with RJ, the particle size increased to 103.7 nm (Figure 1B), indicating the successful incorporation of RJ into the nanostructures. The zeta potential of the free LDH nanoparticles was measured at -25 ± 3 mV, suggesting good colloidal stability, and shifted to -20 ± 2 mV after RJ loading, likely due to surface interactions between RJ and the nanoparticle matrix.

**Figure 1:**
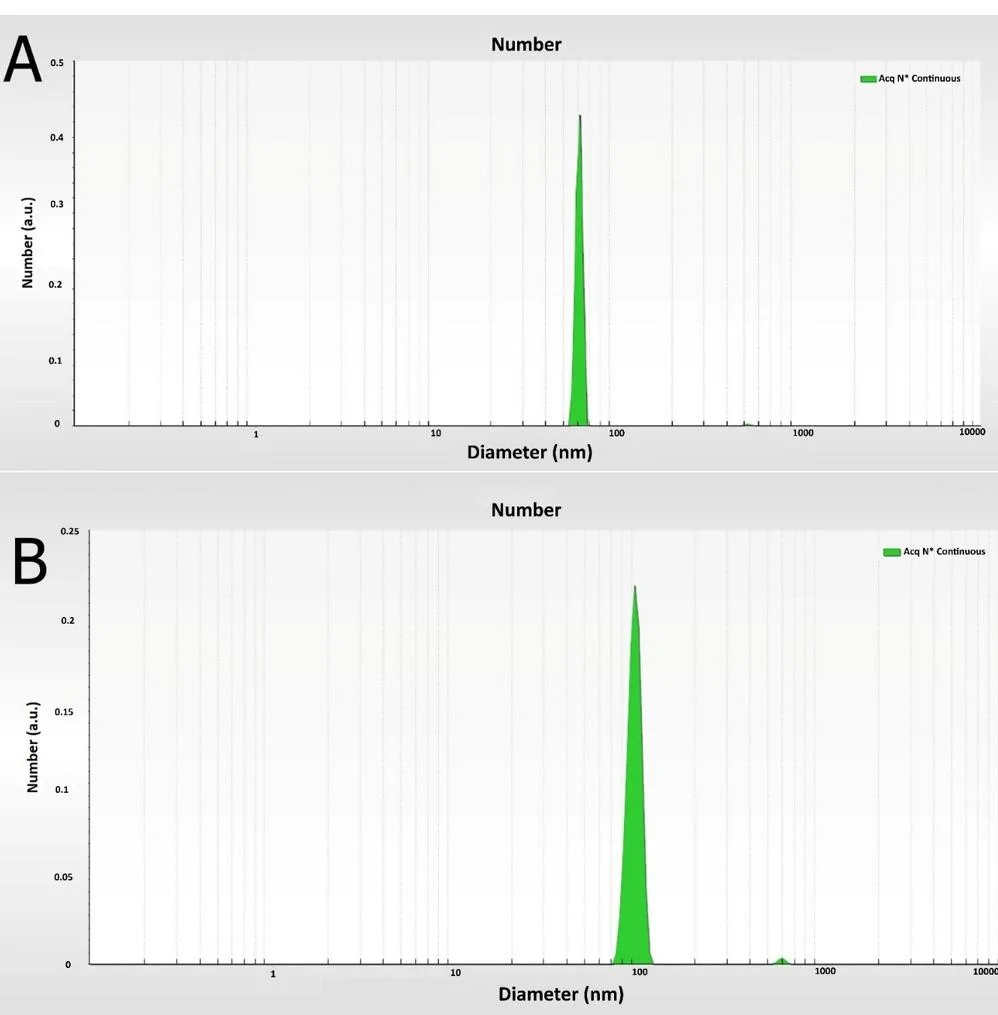
The average diameter of nanoparticles measured by DLS. (A) Size distribution of free LDH nanoparticles, with an average diameter of 61.8 nm. (B) Size distribution of royal jelly-loaded nanoparticles, with an average diameter of 103.7 nm

 Scanning Electron Microscopy (SEM) images (Figure 2) provided further insight into the structural properties of free and RJ-loaded LDHnanostructures. Free-NP exhibited a porous, irregular structure (Figure 2A). At the same time, RJ-loaded LDH nanoparticles (NP-RJ) displayed a more compact, layered morphology with a rough surface texture (Figure 2B), indicating successful encapsulation without significant aggregation.

**Figure 2:**
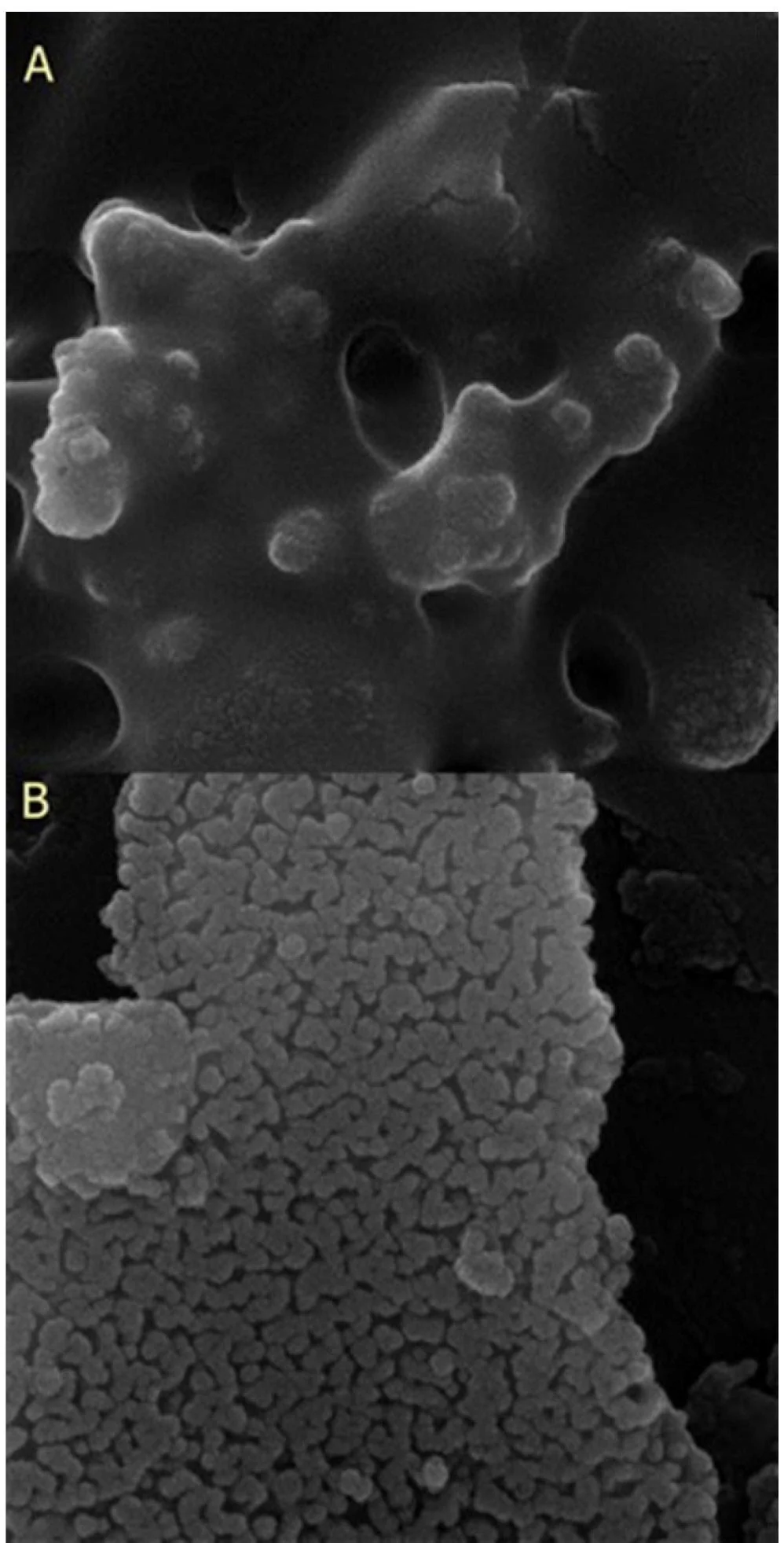
The morphology of the nanoparticles was examined using Scanning Electron Microscopy

 SEM images of (A) free royal jelly (free-RJ), showing a porous and irregular structure, and (B) royal jelly-loaded LDH nanoparticles (NP-RJ), displaying a compact, layered morphology with a rough surface texture.

 Analysis of FTIR data (Figure 3) confirmed the presence of RJ within the LDH nanostructures. The spectrum of Free-RJ (curve A) showed characteristic peaks at 1650 cm⁻¹ (amide I band) and 1540 cm⁻¹ (amide II band), corresponding to its protein content. The spectrum of RJ-loaded LDH (NP-RJ) nanostructures (curve B) retained these amide peaks alongside LDH-specific peaks at 3400 cm⁻¹ (O-H stretching), 1350 cm⁻¹ (carbonate stretching), and 600–800 cm⁻¹ (metal-oxygen vibrations). A slight shift in the O-H stretching peak to 3380 cm⁻¹ suggested hydrogen bonding interactions between RJ and the LDH layers, confirming successful loading.

**Figure 3:**
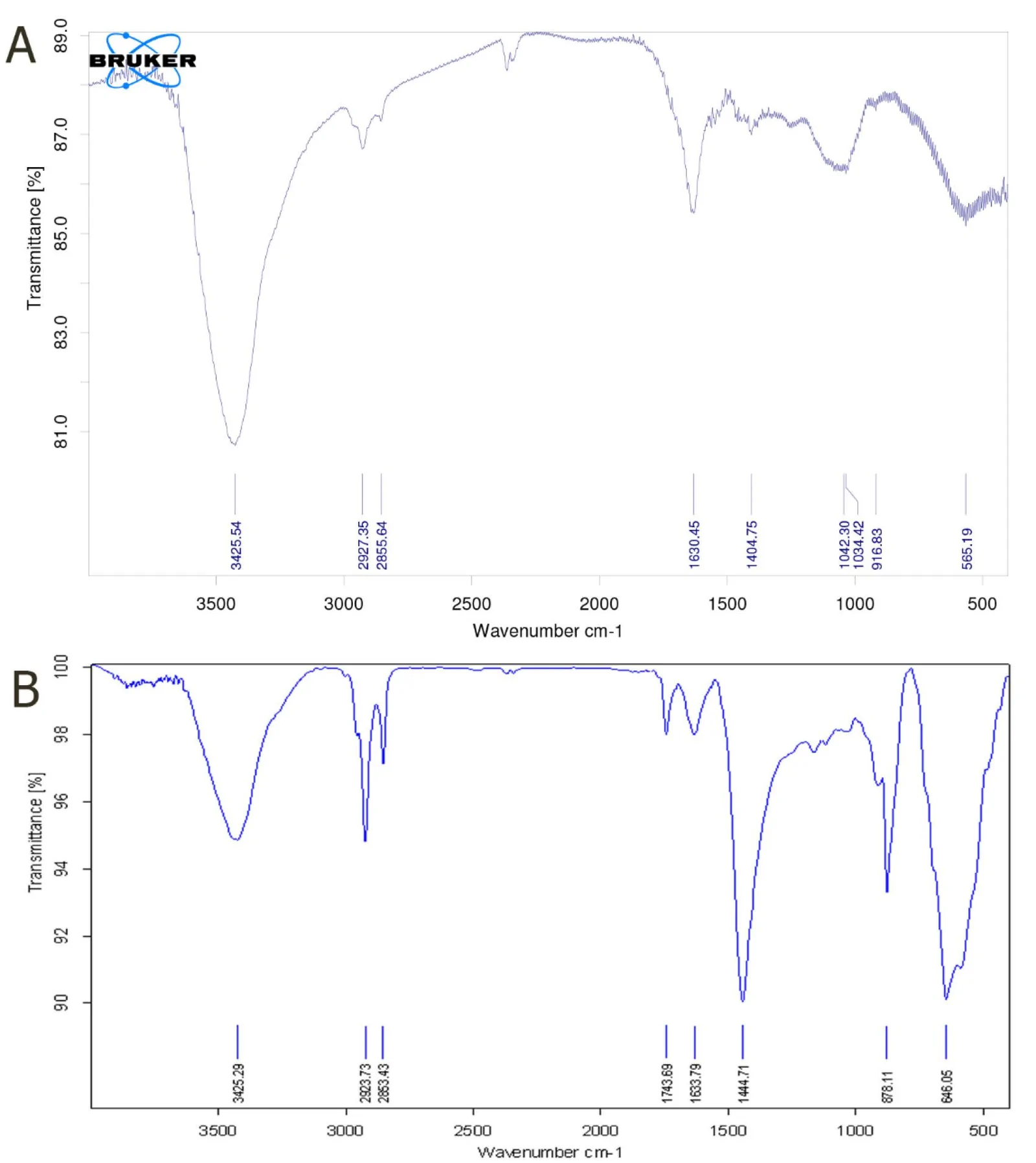
FTIR spectra of (A) free royal jelly (Free-RJ) and (B) royal jelly-loaded LDH nanostructures (NP-RJ), confirming the successful incorporation of royal jelly through the appearance of amide I and II bands

###  In Vitro Release of Royal Jelly from Gel Formulation

 The release profile of RJ from the gel formulation was evaluated using a Franz diffusion cell (Figure 4). The cumulative release of RJ showed a sustained release pattern over 240 minutes. At 15 minutes, approximately 12 ± 2% of RJ was released, which increased to 58 ± 5% by 120 minutes and reached 78 ± 6% by 240 minutes (Figure 4). This sustained release behavior can be attributed to the encapsulation of RJ within the LDH nanostructures and the gel matrix, which provided a controlled diffusion barrier.

**Figure 4:**
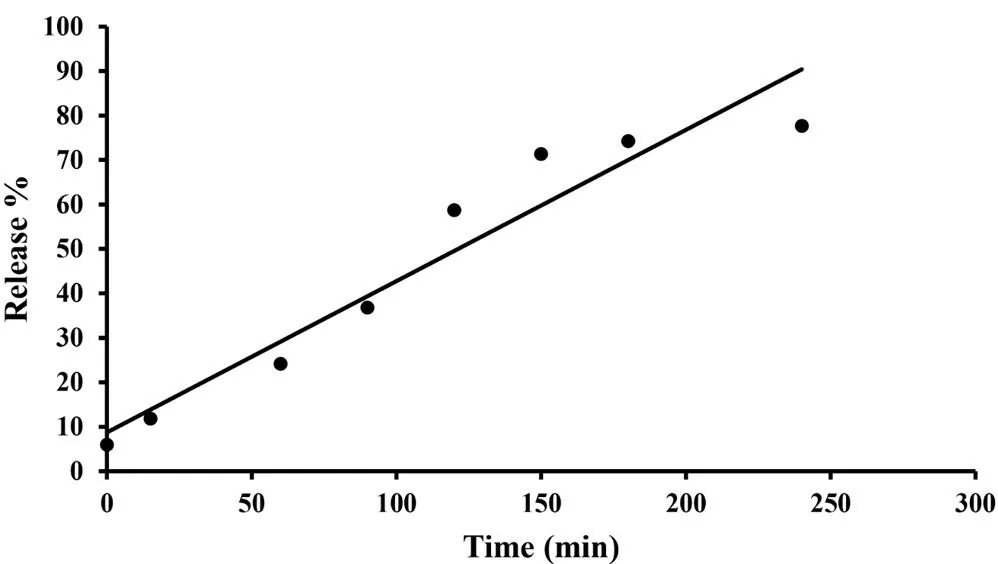
The cumulative release profile of royal jelly from the gel formulation over 240 minutes, measured using a Franz diffusion cell at 37°C, showed a sustained release pattern with 78 ± 6% release by 240 minutes

###  Cytokine and IgE Levels in Serum

 To assess the effect of RJ and its nanoformulations on AD disease, the serum levels of IL-1β, IL-18, and IgE were measured using ELISA technique (Figures 5 and 6). *In vitro* analysis of sera collected from AD mice showed significantly decreased secretion of the pro-inflammatory cytokine IL-18 in the Free-RJ treated group compared to the control group (*P* < 0.05).

**Figure 5:**
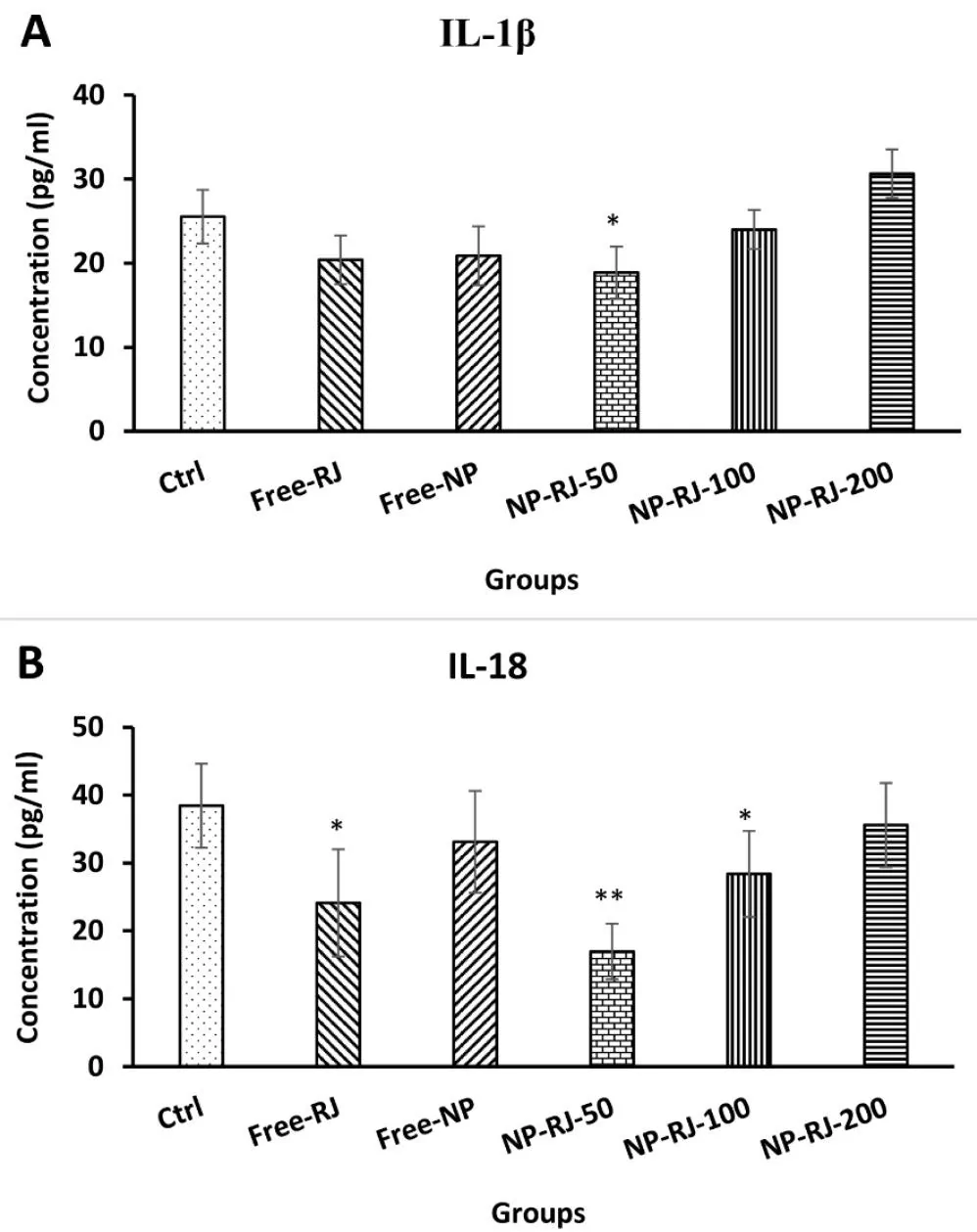
Serum levels of (A) IL-1β and (B) IL-18 in different treatment groups were measured by ELISA. Data are presented as mean ± SEM (n = 6). *P < 0.05, and **P < 0.01, compared to the control group (Ctrl)

**Figure 6:**
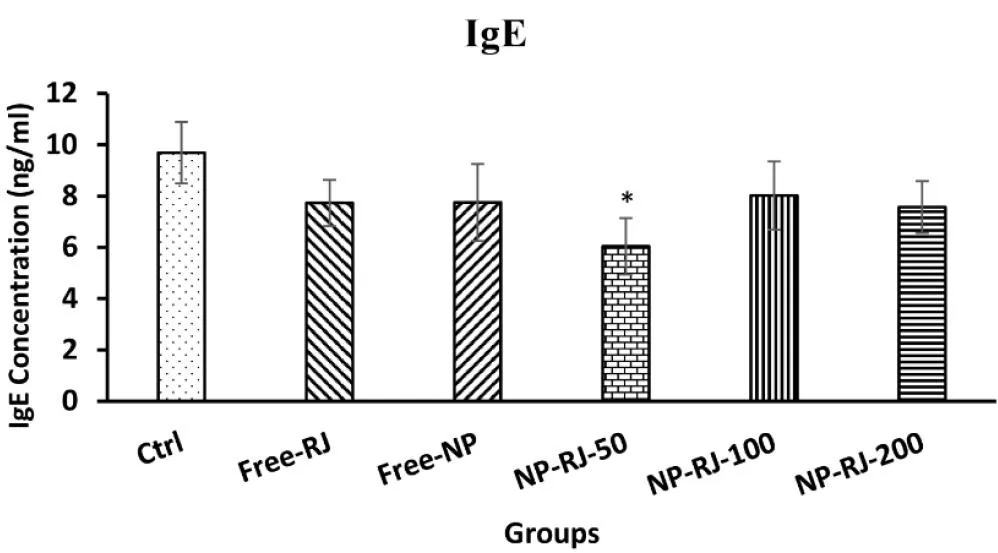
Serum levels of IgE in different treatment groups, measured by ELISA. Data are presented as mean ± SEM (n = 6). *P < 0.05 compared to the control group (Ctrl)

 Treatment with RJ-loaded LDH nanostructures (RJ-NP) showed a reduction in cytokine levels. NP-RJ-50 significantly reduced both IL-1β and IL-18 (*P* < 0.05, *P* < 0.01, respectively). NP-RJ-100 only significantly reduced IL-18 (*P* < 0.05), and NP-RJ-200 showed no significant reduction in either cytokine.

 In addition, to evaluate the therapeutic activity of RJ and its nanoformulation on the level of IgE, ELISA was performed. As shown in Figure 6, only NP-RJ-50 significantly reduced IgE (*P* < 0.05). NP-RJ-100 and NP-RJ-200 showed no effect.

###  Gene Expression Analysis of Inflammasome and Related Molecules 

 The expression levels of inflammasome (NLRP3) and its downstream molecules (Caspase-1, IL-1β, and IL-18) in spleen cells were analyzed using RT-qPCR (Figure 7).

**Figure 7:**
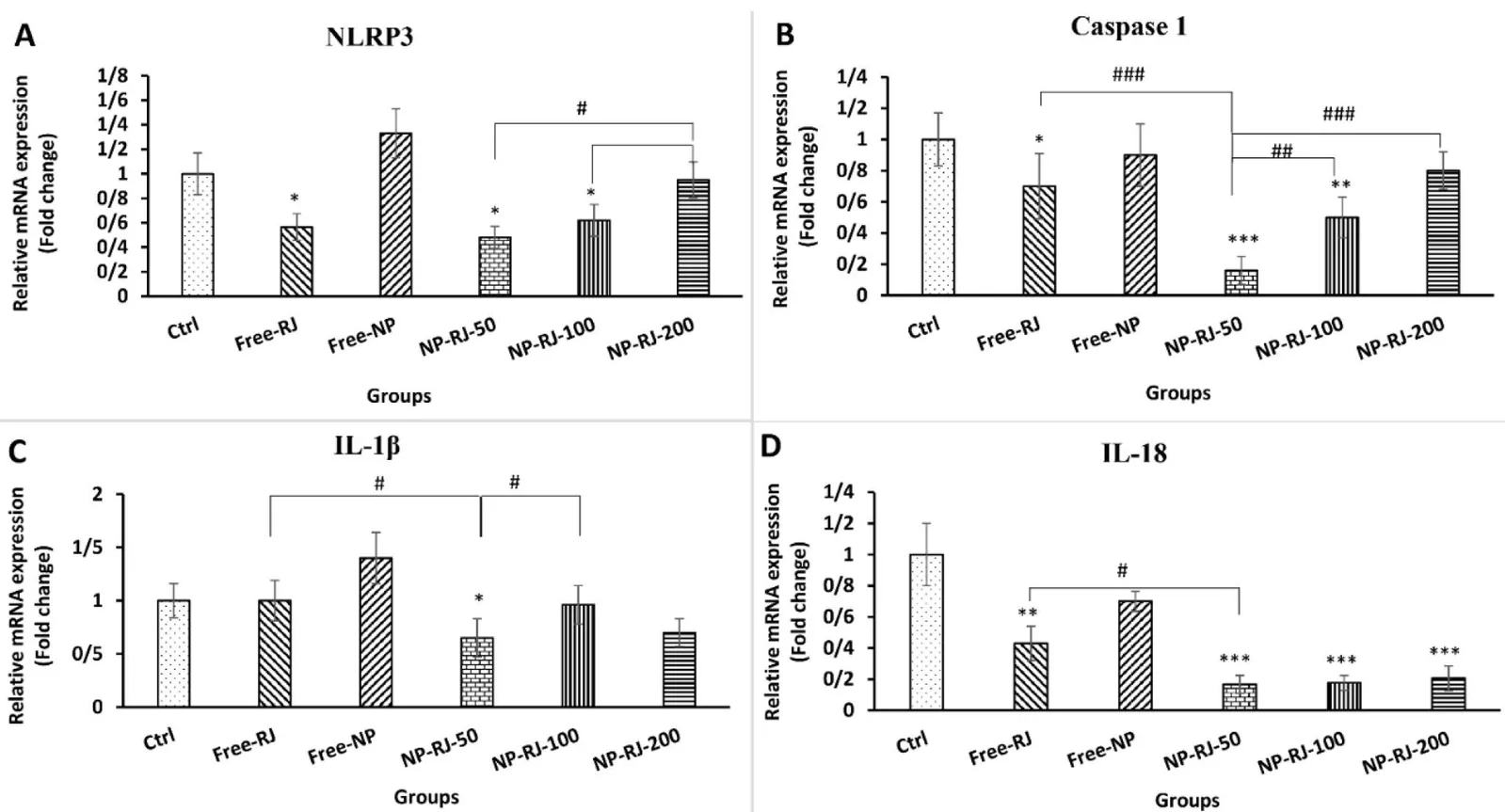
Relative mRNA expression levels of (A) NLRP3, (B) Caspase-1, (C) IL-1β, and (D) IL-18 in spleen cells of different treatment groups, normalized to β-actin. Data are presented as mean ± SEM (n = 6). *P < 0.05, **P < 0.01, ***P < 0.001 compared to the control group (Ctrl)

 Treatment with Free-RJ reduced the expression of NLRP3 (Figure 7A, *P* < 0.05), Caspase-1 (Figure 7B, *P* < 0.05), and IL-18 (Figure 7D, *P* < 0.01) compared to the control group. Unloaded LDH nanostructures (Free-NP) showed no significant effect on genes expression. In contrast, RJ-loaded LDH nanostructures at 50 mg/kg (NP-RJ-50) exhibited a reduction in the expression of NLRP3, Caspase-1, IL-1 β, and I-18 (*P* < 0.05 for NLRP3, and IL-1 β, and *P* < 0.001 for Caspase-1, and IL-18).

 Furthermore, NP-RJ-100 treatment also revealed a significant effect on the of NLRP3, Caspase-1, and I-18 levels as compared with control group.

 NP-RJ-200 shows less consistent effects, with only IL-18 showing a significant reduction.

###  Histopathological Analysis

 Histopathological examining skin samples stained with hematoxylin and eosin (H&E) revealed significant differences between groups (Figure 8). The control group (Figure 8A) exhibited marked epidermal thickening, hyperkeratosis, and inflammatory cell infiltration, consistent with AD. Free-RJ treatment (Figure 8B) moderately reduced epidermal thickness and inflammation, though inflammatory cell infiltration was still evident. Treatment with Free-NP (Figure 8C) showed no notable improvement, with persistent inflammation and epidermal thickening.

**Figure 8:**
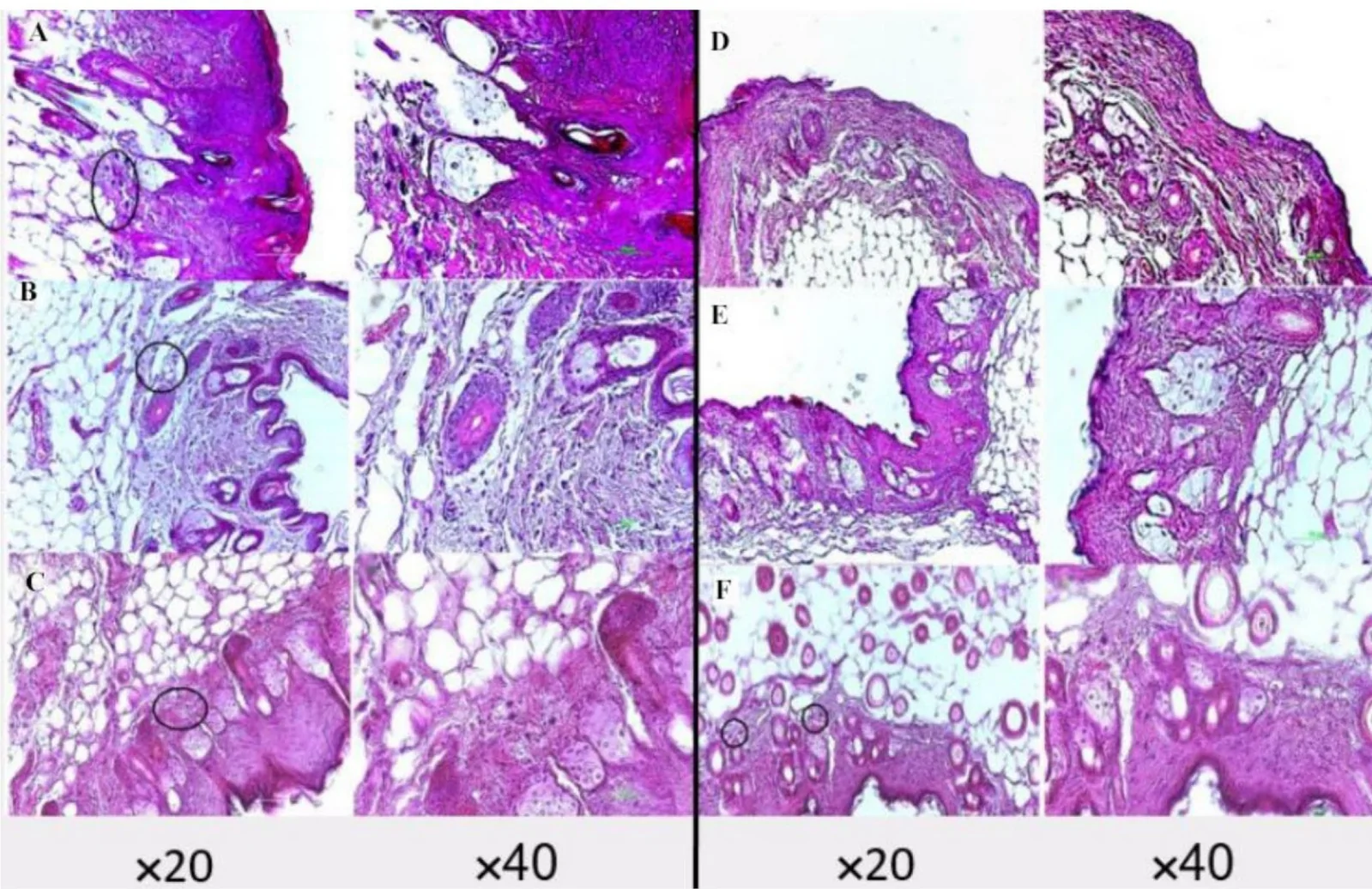
H&E-stained skin sections at 20 × and 40 × magnifications from (A) disease control group (Ctrl), showing epidermal thickening and inflammatory cell infiltration; (B) free royal jelly treatment group (Free-RJ); (C) unloaded LDH nanoparticle treatment group (Free-NP); (D) 50 mg/kg royal jelly-loaded LDH nanoparticle treatment group (NP-RJ-50); (E) 100 mg/kg royal jelly-loaded LDH nanoparticle treatment group (NP-RJ-100); and (F) 200 mg/kg royal jelly-loaded LDH nanoparticle treatment group (NP-RJ-200). The nano formulation groups show a dose-dependent reduction in epidermal thickening and inflammation, with marked areas indicating inflammatory cell infiltration

 RJ-loaded LDH nanostructures (NP-RJ-50, NP-RJ-100, and NP-RJ-200) demonstrated a dose-dependent improvement in skin histology (Figures 8D–8F). The NP-RJ-50 group (Figure 8D) showed a reduction in epidermal thickness and inflammatory cell infiltration compared to the control. The NP-RJ-100 and NP-RJ-200 groups (Figures 8E, and F) improved further, with less inflammation and reduced epidermal thickening, and significantly decreased inflammatory cell infiltration.

## Discussion

 AD is defined as a chronic inflammatory skin disorder marked by symptoms such as itching, redness, scaling, cracking, and ulceration.^2,28^ Conventional treatments, such as corticosteroids, modulate immune responses to reduce symptoms but do not offer a cure and are associated with adverse effects during long-term application. This has spurred interest in safer, more effective therapeutic options.^29^ RJ, a natural secretion from honeybees used to nourish the queen and larvae, has been recognized for its anti-inflammatory and immunomodulatory properties.^10,11^ Studies have demonstrated its potential to mitigate inflammation and support skin health, yet its clinical application is limited by poor stability and bioavailability.^12,14^ Nanotechnology has emerged as a transformative approach to address these limitations, enhancing drug efficacy, reducing required doses, minimizing side effects, and improving stability.^30,31^ LDH nanoparticles, in particular, offer advantages such as appropriate size, solubility, low cytotoxicity, enhanced permeability across cellular and tissue barriers, and improved drug delivery.^32^ The layered structure of LDH enables the intercalation and controlled release of bioactive compounds, reducing the need for high doses.^33^ This system has effectively delivered anti-inflammatory agents, as evidenced by Yu et al, who reviewed LDH-based hybrids like LDH–indomethacin for neuroinflammatory conditions, drug delivery, and imaging.^34^ Motivated by these developments, we explored the therapeutic potential of RJ-loaded LDH nanostructures at various concentrations in a DNCB-induced AD mouse model.

 In this study, we synthesized an RJ-LDH conjugate and assessed its efficacy in a murine AD model. Both Free-RJ and RJ-loaded LDH nanostructures were evaluated for their therapeutic and immunological effects. Our findings revealed that RJ, primarily when delivered via LDH nanostructures, significantly reduced inflammatory cell infiltration into the skin and alleviated cutaneous symptoms such as dryness, itching, and redness. This effect was most pronounced in groups treated with RJ-loaded LDH at 50 and 100 mg/kg (NP-RJ-50 and NP-RJ-100), suggesting that the nanoformulation enhances RJ’s efficacy compared to its free form. These results align with prior research, such as Yamaura et al.’s study, which demonstrated that topical RJ reduced pruritus in a murine model of allergic contact dermatitis.^35^ Furthermore, Gu et al. reported that 10-HDA, a key RJ component, upregulates filaggrin expression in a 3D epidermal model, reinforcing RJ’s role in supporting skin barrier function, a critical aspect of AD management.^36^

 Another investigation on UV-irradiated mice showed that royal jelly treatment increased total filaggrin (including profilaggrin and filaggrin) and the level of epidermal hydration.^37^

 Recently, research has shown that 10-HDA induces filaggrin and collagen production, and subsequently increases stratum corneum thickness in human skin models.^38^

 Researchers documented that filaggrin is a structural protein in the stratum corneum of the epidermis and an essential factor in the development and stability of the skin barrier. Studies have also shown that, mutations in *FLG*, the gene of filaggrin, are responsible for developing AD and other atopic diseases, and exacerbate certain conditions.^39^

 According to the aforementioned studies, it seems that royal jelly plays an immunomodulatory role in AD disease via the production of filaggrin and promoting skin barrier function.

 Furthermore, the possible mechanism involved in AD pathogenesis is inflammation-driven skin barrier dysfunction, with the inflammasome complex playing a central role in its pathogenesis.^40^ Inflammasomes are cytosolic receptors that detect pathogens and endogenous danger signals, triggering innate immune responses. Comprising multiprotein complexes such as NLRP1, NLRP3, NLRC4, and AIM2, they activate Caspase-1, which then processes pro-inflammatory cytokines like pro-IL-1β, pro-IL-18, and pro-IL-33 into their active forms.^41^ Our study found that RJ, particularly in nanoform at 50 and 100 mg/kg, significantly reduced NLRP3 expression in spleen cells, while the nanoformulated RJ also decreased Caspase-1 expression. These findings indicate that RJ modulates inflammasome-mediated inflammation, with the nanoformulation amplifying this effect.

 Although no previous studies have directly investigated RJ’s impact on inflammasome components in AD, related research supports our observations. For instance, Bahaaldin-Beygi et al. reported that RJ reduced NLRP1 expression in the blood of chronic hepatitis B patients.^42^ Similarly, You et al. indicated that 10-HDA inhibited the NLRP3-IL-1β pathway and the TNF-α/NF-κB axis in LPS-induced neuroinflammation in mice.^43^ We examined RJ’s mechanisms to explore further its effects on downstream inflammasome molecules, IL-1β and IL-18. Our results showed that RJ reduced both gene and protein levels of IL-18 in AD mice, with the most significant reduction observed in the NP-RJ-50 group. Excessive IL-1β and IL-18 production are implicated in AD and other inflammatory conditions.^44^ Our findings are in parallel with other studies, such as Taniguchi et al, found that oral RJ reduced IFN-γ in NC/Nga mice with dermatitis-like lesions.^45^ Additional research on renal inflammation and valproic acid-induced organ damage in rats showed RJ decreases IL-1β, and IL-18, and TNF-α levels.^46,47^ Kohno et al.’s *in vitro* study further confirmed that RJ suppressed IL-1 β, TNF-α, and IL-6 in LPS- and IFN-γ-stimulated macrophages without cytotoxicity.^48^ These collective results suggest that RJ’s therapeutic action in AD involves suppressing inflammasome activity and its downstream mediators.^49^

 We also evaluated RJ’s effect on IgE, a key antibody in AD-related allergic responses. Elevated IgE levels drive allergic inflammation, prompting efforts to develop treatments that mitigate its activity. Our study indicated treatment with RJ, particularly at 50 mg/kg in nanoform (NP-RJ-50), reduced IgE levels, though reductions in Free-RJ and higher nanoform doses (100 and 200 mg/kg) were not statistically significant. While no prior studies have specifically examined RJ’s effect on IgE in AD, Xu et al. reported that RJ inhibited mast cell activation via IgE which resulted in reduced histamine and hexosaminidase release by mast cells.^50^ The study also reported RJ improved allergic symptoms in passive-cutaneous anaphylaxis and ovalbumin-induced allergy in animal models. However, conflicting evidence exists, with Thien et al. suggesting that RJ at high doses or prolonged use may increase IgE levels or trigger anaphylaxis in some cases.^51^ Our data indicate that RJ at 50 mg/kg in nanoform effectively reduces IgE, while higher doses showed no significant effect.

 Histopathological analysis revealed a marked improvement in skin architecture in the RJ-loaded LDH groups, with reduced epidermal thickening and inflammatory cell infiltration, particularly at the higher doses (NP-RJ-100, NP-RJ-200).

 In our study, the NP-RJ-50 formulation (low dose) demonstrated optimal therapeutic outcomes in terms of cytokine modulation and IgE reduction. While higher doses (NP-RJ-100 and NP-RJ-200) showed slightly better histological improvement, they were associated with less favorable immunological profiles, suggesting that excessive loading of royal jelly may alter local immune responses, while royal jelly at low dose has a better systemic effect on immune responses.

 Specifically, we propose that higher RJ concentrations may exert mild pro-inflammatory or oxidative effects, as reported in previous studies on bee-derived bioactive compounds.^52^ These effects might counterbalance the anti-inflammatory benefits observed in mid-range doses. Therefore, the NP-RJ-50 formulation represents the most balanced therapeutic window—sufficient to suppress inflammasome activation while avoiding overstimulation of immune pathways.

 These results align with the molecular and immunological data, suggesting that the nanoformulation not only mitigates inflammation at the molecular level but also translates into tangible improvements in skin pathology. The superior efficacy of the nanoformulation compared to Free-RJ can be attributed to several factors: (1) the protective effect of LDH against RJ degradation, (2) the sustained release profile that ensures prolonged exposure, and (3) the enhanced penetration of nanoparticles through the compromised skin barrier in AD.

 Despite these promising results, several limitations should be acknowledged. The study was conducted in a mouse model, and the translation of these findings to human AD requires further investigation, given differences in skin physiology and immune responses between species. Additionally, the long-term safety and stability of the RJ-loaded LDH formulation need to be evaluated, particularly for chronic use in AD management. Future studies should also explore the mechanisms underlying the interaction between RJ and inflammasome pathways in greater detail, potentially using proteomic or transcriptomic approaches to identify additional targets.

## Conclusion

 This study, for the first time, investigated the effects of RJ in both its free form and loaded into LDH nanostructures as a treatment for an animal model of AD. The results demonstrated that RJ significantly reduced inflammatory cell infiltration into the skin and alleviated clinical symptoms of the disease. This reduction was accompanied by downregulated expression of NLRP3 and Caspase-1 and reduced levels of IL-1β and IL-18 in the spleen of affected mice. Based on these findings, it appears that the mechanism of action of these therapeutic compounds in AD involves modulation of the inflammasome pathway and its downstream molecules. Furthermore, the study revealed that loading RJ into LDH nanoparticles resulted in a synergistic effect, enhancing RJ’s pharmacological properties and therapeutic efficacy *in vivo*. Comparison of varying nanoparticle doses with Free-RJ indicated that a lower dose of the nano formulation was required to achieve equivalent or superior efficacy. Among the nanoparticles tested, NP-RJ-50 exhibited a superior therapeutic effect compared to other nanoparticle formulations and Free-RJ. Thus, these findings suggest that RJ-loaded LDH nanostructures hold significant potential as novel therapeutic formulations for treating atopic dermatitis.

## Competing Interests

 The authors declare that they have no known competing financial interests or personal relationships that could have appeared to influence the work reported in this paper.

## Data Availability Statement

 The data that support the findings of this study are available from the corresponding author upon reasonable request.

## Ethical Approval

 The research was approved by the Ethics Committee of Kerman University of Medical Sciences (approval code: IR.KMU.AH.REC.1399.175, approval number 99000771).
